# *HLA* class II alleles may influence susceptibility to adult dermatomyositis and polymyositis in a Han Chinese population

**DOI:** 10.1186/1471-5945-14-9

**Published:** 2014-06-04

**Authors:** Xiang Gao, Lei Han, Lan Yuan, Yongchen Yang, Guimei Gou, Hengjuan Sun, Ling Lu, Liming Bao

**Affiliations:** 1 The First Department of Health Care, Weifang People’s Hospital, Shandong, China; 2 Departments of Rheumatology and Occupational Medicine, Huashan Hospital of Fudan University, Shanghai, China; 3 Center for Clinical Molecular Medicine; Ministry of Education Key Laboratory of Child Development and Disorders, Key Laboratory of Pediatrics in Chongqing, Chongqing, China; 4 Chongqing International Science and Technology Cooperation Center for Child Development and Disorders, Children’s Hospital of Chongqing Medical University, Chongqing, China; 5 Shanghai Children’s Hospital, Shanghai Children’s Hospital Affiliated to Shanghai Jiao Tong University School of Medicine, Shanghai, China; 6 Department of Pathology, Geisel School of Medicine at Dartmouth College, Lebanon, New Hampshire, USA

**Keywords:** Polymyositis, Dermatomyositis, *HLA*, Susceptibility, Chinese

## Abstract

**Background:**

Polymyositis (PM) and dermatomyositis (DM) are idiopathic inflammatory myopathies. Genetic variability in human leukocyte antigen (*HLA*) genes plays an important role in the pathogenesis of PM and DM. However, few studies on the subject in Chinese populations have been reported thus far.

**Methods:**

We studied the influence of *HLA* polymorphisms on DM and PM susceptibility by analyzing *HLA-DRB1*, *HLA*-*DQA1*, and *HLA*-*DQB1* alleles in 71 adult DM patients, 20 adult PM patients, and 113 controls in a Han Chinese population.

**Results:**

A positive association was found between HLA-DQA1*0104 and DM (*p* = 0.01; corrected *p* (*p*_corr_) NS; odds ratio (OR) = 2.58; 95% confidence interval (CI): 1.18–5.64), while an inverse correlation was noted between HLA-DQB1*0303 and myositis patients with interstitial lung inflammation (*p* = 0.01; *p*_corr_ NS; OR = 0.25; 95% CI: 0.07–0.73). A positive relationship was also observed between HLA-DRB1*07 and DM (*p* = 0.01; *p*_corr_ NS; OR = 2.26; 95% CI: 1.12–4.59), while HLA-DRB1*03 seems to be protective against DM (*p* = 0.01; *p*_corr_ NS; OR = 0.26; 95% CI: 0.06–0.81). The lung complication was closely associated with HLA-DRB1*04 (*p* = 0.01; *p*_corr_ NS; OR = 2.82; 95% CI: 1.15–6.76) and HLA-DRB1*12 (*p* = 0.02; *p*_corr_ NS; OR = 2.52; 95% CI: 1.02–6.07). The frequency of HLA-DRB1*07 was significantly higher among myositis patients with dysphagia than among controls (*p* = 0.01; *p*_corr_ NS; OR = 4.78; 95% CI: 1.03–24.42). The putative haplotype DRB1*07-DQA1*01-DQB1*02 was positively correlated with DM (*p* = 0.03; *p*_corr_ NS; OR = 2.90; 95% CI: 1.02–8.93) and the lung complication (*p* = 0.02; *p*_corr_ NS; OR = 3.45; 95% CI: 1.04–11.58).

**Conclusions:**

Our results demonstrate that *HLA* alleles may be involved in susceptibility to adult DM and PM in the Han Chinese population.

## Background

Polymyositis (PM) and dermatomyositis (DM) are idiopathic inflammatory myopathies (IIM), a group of autoimmune disorders characterized by inflammation present predominantly in muscle tissues. The chief clinical presentations include symmetric proximal muscle weakness, other organ involvement, and presence of autoantibodies [1]. Although the underlying pathogenesis for PM and DM remains unclear, evidence has suggested that, like many other autoimmune conditions, these disorders likely result from a combination of environmental exposure and genetic susceptibility [2]. Genetic variability in the human leukocyte antigen (*HLA*) genes is thought to play an important role in DM and PM pathogenesis [3,4]. This may be partly attributed to the influence of HLA molecules on T-cell receptor development, peripheral tolerance, and immune response to environmental agents. It has been established that geographic locations and ethnicities may affect susceptibility to autoimmune diseases [5,6]. The HLA-DRB1*0301 and HLA-DQA1*0501 alleles have been reported as risk factors for myositis in Western populations [7,8], whereas DRB1*0803 may increase PM susceptibility among the Japanese population [9]. In studies of 52 DM and PM patients from Northern China, Han *et al.* reported positive associations between the HLA-DRB1*04, HLA-DRB1*07, and HLA-DRB1*12 alleles and DM development [10]. They also observed that HLA-DQB1*0401 is a risk factor for DM and PM [11]. However, the sample sizes of these studies were small; therefore, the possible role of *HLA* class II alleles in myopathies in Chinese patients requires further investigation.

To assess the effect of polymorphisms in *HLA-DRB1*, *HLA*-*DQA1*, and *HLA*-*DQB1* on DM and PM susceptibility, we conducted a study of 91 adult patients with DM or PM and 113 healthy controls in a Han Chinese population. Our results demonstrate that *HLA* class II alleles may influence adult DM and PM susceptibility in the Han Chinese population.

## Methods

### Study subjects

Between August 2009 and March 2012, 71 and 20 patients were diagnosed with DM and PM, respectively, at the Huashan Hospital of Fudan University in Shanghai, China. The patients met probable or definite diagnosis of DM or PM according to the Bohan and Peter [12] criteria [12,13]. Lung lesions were examined by chest computed tomography, and a diagnosis of interstitial lung disease (ILD) or idiopathic interstitial pneumonitis was made by a pulmonologist. All patients were ethnic Han Chinese (25 males and 66 females, median age of 51 ± 15.8 years). One hundred and thirteen healthy Han Chinese subjects (36 males and 77 females, median age of 40.0 ± 8.6 years) with no history of autoimmune disease were enrolled in the study as controls. The study followed protocols set by the Declaration of Helsinki and was approved by the ethics committee of Huashan Hospital. All patients and controls provided written informed consent prior to the study.

### Experiments

DNA was extracted from blood using the Qiagen DNA extraction kit (Qiagen; Hilden, Germany). Low- and high-resolution typing of the *HLA-DRB1*, *HLA*-*DQA1*, and *HLA*-*DQB1* alleles were performed using the polymerase chain reaction (PCR)-sequence-specific primed (SSP) procedure described by Olerup *et al*. [14,15]. Serum was obtained from all patients for detection of myositis-specific antibodies (MSA; anti-Jo-1 autoantibodies) and myositis-associated antibodies (MAA; anti-Ro and anti-La autoantibodies). Autoantibodies were analyzed by an indirect immunofluorescence method using the kits from EUROIMMUN AG (Lubeck, Germany).

### Statistical analysis

The allele, genotype, and haplotype frequencies of the *HLA* loci were compared between the patients and controls using the Fisher exact test or chi-square test, as appropriate. Data were expressed as odds ratios (ORs) with 95% confidence intervals (CIs). A *p-*value ≤ 0.05 was considered statistically significant. In multiple comparisons, *p-*values were adjusted using the Bonferroni correction to give a corrected *p-*value (*p*_corr_). A *p*-value ≤ 0.05 before correction for multiple comparisons was considered as possibly statistically significant. The Hardy–Weinberg equilibrium constant in the control group was confirmed using a chi-square test. Putative haplotypes were inferred from unphased genotype data using the Bayesian statistical method available in the PHASE program v2.1.1 [16]. Putative haplotypes with a frequency higher than 3% among the controls were selected for further comparisons between the patients and controls. Unless otherwise stated, Stata (version 7.0; Stata Corp.; College Station, TX, USA) statistical software was used to perform the statistical analyses.

## Results

Of the 91 patients, 71 had been diagnosed with DM and 20 with PM (66 female and 25 male [2.64:1]). Although most of the patients were positive for antinuclear antibodies, few were positive for either MSA or MAA. The frequencies of MSA and MAA were higher in the PM group than in the DM group (10% vs. 1.4%, *p* = 0.06 for MSA; 20% vs. 5.63%, *p* = 0.05 for MAA). As expected, cutaneous involvement such as rash or cuticular overgrowth was present only in DM patients. Approximately half of the patients had ILD, with a higher prevalence among patients with PM than those with DM (76.5% vs. 37.7%, *p* = 0.04). No significant differences in other clinical features were observed between the two groups (Table 1).

**Table 1:** Clinical features of patients with DM and PM

	**DM n (%) (N = 71)**	**PM n (%) (N = 20)**	***p *** **(DM vs. PM)**
Serologic group			
MSA (anti-Jo-1)	1 (1.4)	2 (10)	0.06
MAA (SSA, SSB or Scl)	4 (5.63)	4 (20)	0.05
Clinical presentations			
Fever	4 (5.6)	1 (5)	0.92
Raynaud’s disease	0.00	1 (5)	0.06
Arthritis	1 (1.4)	0.00	0.59
Interstitial lung disease	26^1^ (37.7)	13^2^ (76.5)	0.04
Palpitations	1 (1.4)	0.00	0.59
Dysphagia	10 (14.1)	4 (20)	0.51
V-sign	26 (36.6)	0.00	n/a
Gottron papules	24 (33.8)	0.00	n/a
Periorbital edematous rash	11 (15.5)	0.00	n/a
Shawl sign	13 (18.3)	0.00	n/a
Cuticular overgrowth	3 (4.2)	0.00	n/a

MSA: myositosis-specific autoantiboides; MAA: myositosis associated autoantibodies;

^1^69 DM patients evaluated; ^2^17 PM patients evaluated; n/a: not applicable.

Genotyping analyses of the *HLA-DQA1* and *HLA-DQB1* loci were performed to study the relationship between the *HLA* class II alleles and susceptibility to DM and PM. Because there are several lines of evidence to suggest that DM and PM may be distinct diseases with different genetic backgrounds [17], we analyzed the influence of the *HLA* alleles on susceptibility to DM and PM individually rather than analyzing the combined DM/PM group (Table 2). Compared with the controls, the frequency of HLA-DQA1*0104 was significantly higher in the DM group than in the PM group (16.42% vs. 8.18%, *p* = 0.01, *p*_*corr*_ NS; OR = 2.58; 95% CI: 1.18–5.74), implying a possible positive correlation between HLA-DQA1*0104 and the risk of DM. Of the *HLA-DQB1* alleles analyzed, only the HLA-DQB1*0303 frequency was lower in patients with ILD than in the controls (6.76% vs. 19.03%, *p* = 0.01, *p*_*corr*_ NS; OR = 0.25; 95% CI: 0.07 − 0.73), implying that patients with this allele are less likely to develop the lung complication. This was further confirmed by the fact that the HLA-DQB1*0303 frequency was lower among those who developed ILD (*p* < 0.01; OR = 0.19; 95% CI: 0.038–0.37) than those who did not develop ILD. *HLA-DRB1* typing analysis showed that compared to controls, DM patients had a considerably lower prevalence of HLA-DRB1*03 (3.08% vs. 11.27%, *p* = 0.01, *p*_*corr*_ NS; OR = 0.26; 95% CI: 0.06–0.81) and a higher frequency of HLA-DRB1*07 (20.77% vs. 13.24%, *p* = 0.01, *p*_*corr*_ NS, OR = 2.26, 95% CI, 1.12–4.59). Both HLA-DRB1*04 and HLA-DRB1*12 alleles are likely linked to the lung disease (*p* = 0.01, *p*_*corr*_ NS; OR = 2.82; 95% CI: 1.15–6.76 and *p* = 0.02, *p*_*corr*_ NS; OR = 2.52; 95% CI: 1.02–6.07, respectively). A similar effect of HLA-DRB1*04 on ILD development was also observed when the allele frequencies between patients with and without the lung complication were compared (*p* = 0.026; OR = 3.32; 95% CI: 1.22–9.08). Comparison of HLA-DRB1*12 allele frequencies between myositis patients with and without ILD showed a similar trend of being higher among those with ILD than among those without, although the difference between the two groups was not statistically significant (*p* = 0.35; OR = 1.59; 95% CI: 0.63–4.00). This is likely attributable to the small numbers in the groups examined. Moreover, the HLA-DRB1*07 allele showed a possible influence on the development of esophageal/muscle complications (30.00% vs. 13.24%, *p* = 0.01, *p*_*corr*_ NS; OR = 4.78; 95% CI: 1.03–24.42). A similar trend for a higher HLA-DRB1*07 allele frequency was also noted among patients who had dysphagia as compared to those who did not (*p* = 0.057; OR = 3.55; 95% CI: 0.91–13.82).

**Table 2:** ***HLA-DQA1*, *HLA-DQB1, *** **and *HLA-DRB1 *** **allele frequencies in PM and DM patients and controls**

**Loci/alleles**	**DM**	**PM**	**Myositis with ILD**	**Myositis with dysphagia**	**Control**	**Control vs. indicated patients ( *p *; OR (95% CI))**			
	**n (%)**	**n (%)**	**n (%)**	**n (%)**	**n (%)**	**DM**	**PM**	**Myositis with ILD**	**Myositis with dysphagia**
*HLA-DRB1*	N = 65	N = 19	N = 37	N = 10	N = 113				
*01	1 (0.77)	0 (0.00)	1 (1.35)	0 (0.00)	5 (2.45)	n/s	n/a	n/s	n/a
*03	4 (3.08)	4 (10.53)	4 (5.41)	1 (5.00)	23 (11.27)	0.01^1^; 0.26	n/s	n/s	n/s
						(0.06–0.81)			
*04	17 (13.08)	6 (15.79)	15 (20.27)	2 (10.00)	22 (10.78)	n/s	n/s	0.01^1^; 2.82	n/s
								(1.15–6.76)	
*07	27 (20.77)	1 (2.63)	14 (18.92)	6 (30.00)	27 (13.24)	0.01^1^; 2.26	n/s	n/s	0.01^1^; 4.78
						(1.12–4.59)			(1.03–24.42)
*08	13 (10.00)	4 (10.53)	7 (9.46)	2 (10.00)	17 (8.33)	n/s	n/s	n/s	n/s
*09	20 (15.38)	2 (5.26)	3 (4.05)	3 (15.00)	26 (12.75)	n/s	n/s	n/s	n/s
*10	2 (1.54)	1 (2.63)	2 (2.70)	0 (0.00)	3 (1.47)	n/s	n/s	n/s	n/a
*11	3 (2.31)	3 (7.89)	2 (2.70)	0 (0.00)	9 (4.41)	n/s	n/s	n/s	n/a
*12	20 (15.38)	7 (18.42)	14 (18.92)	1 (5.00)	22 (10.78)	n/s	n/s	0.02^1^; 2.52	n/s
								(1.02–6.07)	
*13	7 (5.38)	1 (2.63)	2 (2.70)	1 (5.00)	8 (3.92)	n/s	n/s	n/s	n/s
*14	2 (1.54)	3 (7.89)	1 (1.35)	2 (10.00)	9 (4.41)	n/s	n/s	n/s	n/s
*15	10 (7.69)	4 (10.53)	7 (9.46)	1 (5.00)	29 (14.22)	n/s	n/s	n/s	n/s
*16	4 (3.08)	2 (5.26)	2 (2.70)	1 (5.00)	4 (1.96)	n/s	n/s	n/s	n/s
*HLA-DQA1*	N = 67	N = 20	N = 39	N = 12	N = 110				
*0101	9 (6.72)	0 (0.00)	5 (6.41)	1 (4.17)	11 (5.00)	n/s	n/a	n/s	n/s
*0102	18 (13.43)	10 (25.00)	16 (20.51)	4 (16.67)	44 (20.00)	n/s	n/s	n/s	n/s
*0103	26 (19.40)	6 (15.00)	10 (12.82)	6 (25.00)	28 (12.73)	n/s	n/s	n/s	n/s
*0104	22 (16.42)	4 (10.00)	11 (14.10)	4 (16.67)	18 (8.18)	0.01^1^; 2.58	n/s	n/s	n/s
						1.18–5.64)			
*0201	1 (0.75)	2 (5.00)	1 (1.28)	0 (0.00)	1 (0.45)	n/s	n/s	n/s	n/a
*0301	44 (32.84)	9 (22.50)	26 (33.33)	9 (37.50)	85 (38.64)	n/s	n/s	n/s	n/s
*0302	1 (0.75)	0 (0.00)	0 (0.00)	0 (0.00)	1 (0.45)	n/s	n/a	n/a	n/a
*0401	1 (0.75)	2 (5.00)	2 (2.56)	0 (0.00)	3 (1.36)	n/s	n/s	n/s	n/a
*0501	11 (8.21)	6 (15.00)	6 (7.69)	0 (0.00)	28 (12.73)	n/s	n/s	n/s	n/a
*0507	1 (0.75)	0 (0.00)	0 (0.00)	0 (0.00)	0 (0.00)	n/a	n/a	n/a	n/a
*0601	0 (0.00)	1 (2.50)	1 (1.28)	0 (0.00)	1 (0.45)	n/a	n/s		
*HLA-DQB1*	N = 67	N = 17	N = 37	N = 10	N = 113				
*0201	31 (23.48)	6 (16.67)	16 (21.62)	5 (25.00)	44 (19.47)	n/s	n/s	n/s	n/s
*0301	20 (15.15)	9 (25.00)	15 (20.27)	0 (0.00)	35 (15.49)	n./s	n/s	n/s	n/a
*0302	9 (6.82)	2 (5.56)	7 (9.46)	1 (5.00)	12 (5.31)	n/s	n/s	n/s	n/s
*0303	24 (18.18)	2 (5.56)	5 (6.76)	5 (25.00)	43 (19.03)	n/s	n/s	0.01^1^; 0.25	n/s
								(0.07–0.73)	
*0401	6 (4.55)	2 (5.56)	6 (8.11)	1 (5.00)	7 (3.10)	n/s	n/s	n/s	n/s
*0402	0 (0.00)	1 (2.78)	1 (1.35)	0 (0.00)	1 (0.44)	n.a.	n/s	n/s	n/a
*0501	1 (0.76)	2 (5.56)	3 (4.05)	0 (0.00)	8 (3.54)	n/s	n/s	n/s	n/a
*0502	5 (3.79)	2 (5.56)	5 (6.76)	0 (0.00)	7 (3.10)	n/s	n/s	n/s	n/a
*0503	4(3.03)	1 (2.78)	0 (0.00)	2 (10.00)	8 (3.54)	n/s	n/s	n/a	n/s
*0601	20 (15.15)	7 (19.44)	11 (14.86)	3 (15.00)	33 (14.60)	n/s	n/s	n/s	n/s
*0602	4 (3.03)	2 (5.56)	2 (2.70)	2 (10.00)	16 (7.08)	n/s	n/s	n/s	n/s
*0604	5 (3.79)	0 (0.00)	2 (2.70)	0 (0.00)	5 (2.21)	n/s	n/a	n/s	n/a
*0608	3 (2.27)	0 (0.00)	1 (1.35)	1 (5.00)	7 (3.10)	n/s	n/a	n/s	n/s

n/s: not significant; n/a: not applicable; ^1^*p*corr greater than 0.05; ILD: interstitial lung disease.

We then examined the association between putative haplotypes involving the *DRB1-DQA1-DQB1* loci and susceptibility to DM, PM, lung, and esophageal complications. In this analysis, only the putative haplotypes present in at least 3% of the controls were selected for further study. Of the 12 putative haplotypes selected, the frequency of DRB1*07-DQA1*01-DQB1*02 was higher in the DM group (*p* = 0.03, *p*_*corr*_ NS; OR = 2.90; 95% CI: 1.02–8.93) and in patients with ILD (*p* = 0.02, *p*_*corr*_ NS; OR = 3.45; 95% CI: 1.04–11.58) than in the controls, indicating that this putative haplotype might increase the risk of DM and the lung complication (Table 3). A further comparison of the DRB1*07-DQA1*01-DQB1*02 frequency in patients who developed dysphagia with those who did not showed a trend for a higher frequency of this haplotype among patients with dysphagia, although the difference between the groups was not statistically significant (*p* = 0.23; OR = 2.27; 95% CI: 0.68–7.72). Again, we consider that this result is likely due to the small sample sizes in both groups.

**Table 3:** **Comparisons of frequencies of *HLA *** **class II putative haplotypes between DM and PM and controls**

**Haplotypes**	**DM (N =63)**	**PM (N = 18)**	**Myositis with ILD**	**Myositis with dysphagia**	**Control (N =100)**	**Controls vs. indicated patients ( *p *; OR [95% CI])**			
***DRB1-DQA1-DQB1***	**n (%)**	**n (%)**	**n (%) N = 36**	**n (%), N = 9**	**n (%)**	**DM**	**PM**	**Myositis with ILD**	**Myositis with dysphagia**
03-05-02	2 (1.59)	2 (5.56)	2 (2.78)	0 (0.00)	11 (5.50)	n/s	n/s	n/s	n/s
04-03-03	6 (4.36)	1 (2.78)	5 (6.94)	1 (5.56)	9 (4.50)	n/s	n/s	n/s	n/s
04-03-04	5 (3.97)	2 (5.56)	5 (6.94)	1 (5.56)	6 (3.00)	n/s	n/s	n/s	n/s
07-01-02	12 (9.52)	1 (2.78)	8 (11.11)	2 (11.11)	7 (3.50)	0.03^1^; 2.90	n/s	0.02^1^; 3.45	n/s
						(1.02–8.93)		(1.04–11.58)	
07-03-02	6 (4.76)	0 (0.00)	3 (4.17)	2 (11.11)	11 (5.50)	n/s	n/s	n/s	n/s
08-01-06	13 (10.32)	3 (8.33)	6 (8.33)	2 (11.11)	13 (6.50)	n/s	n/s	n/s	n/s
09-03-03	17 (13.49)	2 (5.56)	3 (4.17)	3 (16.67)	20 (10.00)	n/s	n/s	n/s	n/s
11-05-03	3 (2.38)	1 (2.78)	1 (1.39)	0 (0.00)	7 (3.50)	n/s	n/s	n/s	n/s
12-01-03	11 (8.73)	2 (5.56)	8 (11.11)	0 (0.00)	11 (5.50)	n/s	n/s	n/s	n/s
13-01-06	7 (5.56)	0 (0.00)	2 (2.78)	1 (5.56)	7 (3.50)	n/s	n/s	n/s	n/s
14-01-05	2 (1.59)	1 (2.78)	0 (0.00)	2 (11.11)	7 (3.50)	n/s	n/s	n/s	n/s
15-01-06	7 (5.56)	3 (8.33)	3 (4.17)	1 (5.56)	19 (9.50)	n/s	n/s	n/s	n/s

n/s: not significant; n/a: not applicable; ^1^*p*corr greater than 0.05; ILD: interstitial lung disease.

## Discussion

There are differences in the clinical features of DM and PM between our cohort of Han Chinese patients and Western patients [1,18]. There are more DM cases in our cohort and among Mesoamerican patients, whereas PM is the major subtype among Caucasian patients [19]. Compared to Caucasian patients, our cohort had a higher prevalence of the lung complication [20]. The frequencies of dysphagia were comparable between our cohort and those reported in the West [1,21]. Approximately 30–40% of Caucasian and African American IIM patients are positive for MSA/MAA autoantibodies [19,22]. Although the proportion of patients who were positive for autoantibodies was relatively low in our cohort, it is nonetheless comparable to findings from other studies in Chinese patients [23,24].

There is a growing body of evidence to suggest that differences in the impact of *HLA* class II alleles on the susceptibility to DM and PM may exist among different ethnic groups and geographic locations [19,20,25-27]. It has been well documented that *HLA* class II alleles that form the 8.1 ancestral haplotype (8.1 AH), DRB1*03-DQA1*05-DQB1*02, are closely linked to DM and PM in Western populations [4,7,28]. Previous studies suggest that HLA-DQA1*0501 and HLA-DRB1*0301 may be risk factors for PM, whereas HLA-DQA1*0201 and HLA-DRB1*0401 confer protection against the disease in Caucasians [4,28-30]. Moreover, HLA-DRB1*07 has been reported to protect against PM and IIM in Caucasians and African Americans [4,22,28]. In their study of African American patients with IIM, O’Hanlon *et al.*[22] demonstrated that HLA-DRB1*14 and HLA-DRB1*0301 increase the risk of DM, while HLA-DRB1*0301 influences PM susceptibility. Furuya *et al.*[9,26] found that both HLA-DRB1*0803 and HLA-DQA1*0501 provide an increased risk of DM but a reduced risk of PM in a Japanese population. In a study of 25 Korean patients with IIM, Rider *et al*. [27] reported that HLA-DRB1*14 as a protective factor for DM and PM. To date, most studies on the subject have been focused on Western populations. In their studies of 52 DM and PM patients from Northern China, Han *et al.* reported that HLA-DRB1*04, HLA-DRB1*07, and HLA-DRB1*12 may render an increased risk of DM [10], while HLA-DQB1*0401 may have an impact on IIM susceptibility [11]. Our data demonstrate that the HLA-DQA1*0104 and HLA-DRB1*07 alleles are likely associated with an increased risk of DM, whereas HLA-DRB1*03 may provide protection against DM. Although 8.1 AH is known as a risk factor for IIM among individuals of Northwestern European descent [4,7,28], such an association has not been established in other ethnic groups [25]. DRB1*03-DQA1*05-DQB1*02 is not a major haplotype in our cohort and does not influence DM and PM susceptibility. Our results suggest that the HLA-DRB1*07-DQA1*01-DQB1*02 haplotype may be associated with DM and ILD.

It is interesting to note that HLA-DRB1*07 influences IIM susceptibility among various ethnic groups. In our study of Han Chinese patients, the HLA-DRB1*07 allele in the putative haplotype DRB1*07-DQA1*01-DQB1*02 was found to be a risk factor for DM, but the same allele present in the DRB1*07-DQA1*02-DQB1*02 haplotype was reported to be a protector against IIM in Caucasians and African Americans [4,22,25,28]. These findings imply that HLA-DRB1*07 might have opposite effects on the diseases in different ethnic groups. Similar phenomena have been observed for the effect of HLA-DQA1*0501 on IIM. This allele was reported as a protective factor against IIM in a Japanese population [9] but as a risk factor for IIM among US Caucasians [29]. Although it is unclear what might cause such different effects, possible explanations include referral bias, small sample sizes resulting in nonrepresentative populations, different pathogeneses, various environmental risk triggers around the world, and unidentified genetic loci responsible for disease development.

Furuya *et al*. [26] reported a positive relationship between HLA-DRB1*0405 and anti-aminoacyl-tRNA synthetase autoantibodies in Japanese IIM patients. In addition, O’Hanlon *et al.* found that the HLA-DRB1*0302 allele is closely linked to myositis-specific anti-Mi-2 autoantibodies in DM [4]. In our study, the numbers of patients with MSA and MAA were too small to have sufficient statistical power for further analysis of the relationship between *HLA* alleles and MSA/MAA autoantibodies. It is also noteworthy that a greater frequency of myositis autoantibodies was found among PM patients from our cohort and the cohort reported by Chinoy and colleagues [20]. However, both cohorts were small in size, and in our study we analyzed only a limited number of autoantibodies.

Previous studies suggest that DM and PM may have different pathogeneses. It has been proposed that DM is likely the result of an autoimmune process induced by a humoral response, whereas PM may be caused by a cell-mediated autoimmune process [17]. Thus, it is plausible that DM and PM might have distinctive genetic susceptibilities. Further investigation is warranted to better understand the pathogeneses of these two diseases.

Possible explanations for the differences between the results from our study and those from other studies include the fact that the studies were conducted in different geographic locations with various environmental exposures, the heterogeneity in genetic backgrounds among the different ethnic groups and populations, the number of *HLA* polymorphic alleles analyzed, the different study methodologies used, referral bias, and the age and sex composition of the different study cohorts. The differences in the clinical and immunogenetic features between adult and childhood DM and PM are well documented [8,31].

Our study has some limitations. Although DM and PM are rare conditions and the number of patients in our cohort is the largest compared with similar studies in Chinese patients, it is still a relatively small cohort. It is likely that there are many genes and genetic polymorphisms that may influence DM and PM susceptibility, and we analyzed only a few of them. The data from our study should be validated by further analysis of multiple genes and alleles and their combined impacts on disease susceptibility. Nevertheless, our data shed some light on the genetic susceptibility of adult DM and PM, and may aid in stratifying disease subtypes according to genetic and ethnic backgrounds.

## Conclusions

Our study of adult DM and PM in a Chinese population demonstrated that the putative haplotype DRB1*07-DQA1*01-DQB1*02 and the DRB1*07 and DQA1*0104 alleles are associated with an increased risk of DM, while DRB1*03 is associated with a reduced risk of DM. DQB1*0303 may confer protection against the lung complication, and DRB1*04, DRB1*12, and DRB1*07-DQA1*01-DQB1*02 are associated with an increased risk of developing the lung complication. Furthermore, DRB1*07 is associated with an increased risk of developing dysphagia.

## Abbreviations

PM: Polymyositis; DM: Dermatomyositis; HLA: Human leukocyte antigen; IIM: Idiopathic inflammatory myopathies.

## Competing interests

The authors declare that they have no competing interests.

## Authors’ contributions

LB, LL and XG conceived the study design and manuscript preparation; LB, LL, XG, LY, LH, HS, and YY participated in data interpretation; XG, YY, LH, LY, HS, and GG collected data and conducted experiments and data analysis. All authors read and approved the final manuscript.

## Pre-publication history

The pre-publication history for this paper can be accessed here:

http://www.biomedcentral.com/1471-5945/14/9/prepub

## References

[B1] Mammen AL Dermatomyositis and polymyositis: Clinical presentation, autoantibodies, and pathogenesis Ann N Y Acad Sci 2010 1184 134 153 10.1111/j.1749-6632.2009.05119.x 20146695

[B2] Luppi P Rossiello MR Faas S Trucco M Genetic background and environment contribute synergistically to the onset of autoimmune diseases J Mol Med (Berl) 1995 73 8 381 393 8528740 10.1007/BF00240137

[B3] Thorsby E Lie BA HLA associated genetic predisposition to autoimmune diseases: Genes involved and possible mechanisms Transpl Immunol 2005 14 3–4 175 182 15982560 10.1016/j.trim.2005.03.021

[B4] O'Hanlon TP Carrick DM Targoff IN Arnett FC Reveille JD Carrington M Gao X Oddis CV Morel PA Malley JD Malley K Shamim EA Rider LG Chanock SJ Foster CB Bunch T Blackshear PJ Plotz PH Love LA Miller FW Immunogenetic risk and protective factors for the idiopathic inflammatory myopathies: distinct HLA-A, -B, -Cw, -DRB1, and -DQA1 allelic profiles distinguish European American patients with different myositis autoantibodies Medicine (Baltimore) 2006 85 2 111 127 10.1097/01.md.0000217525.82287.eb 16609350

[B5] Seldin MF Amos CI Ward R Gregersen PK The genetics revolution and the assault on rheumatoid arthritis Arthritis Rheum 1999 42 6 1071 1079 10.1002/1529-0131(199906)42:6<1071::AID-ANR1>3.0.CO;2-8 10366098

[B6] McLeod R Buschman E Arbuckle LD Skamene E Immunogenetics in the analysis of resistance to intracellular pathogens Curr Opin Immunol 1995 7 4 539 552 10.1016/0952-7915(95)80100-6 7495519

[B7] Arnett FC Targoff IN Mimori T Goldstein R Warner NB Reveille JD Interrelationship of major histocompatibility complex class II alleles and autoantibodies in four ethnic groups with various forms of myositis Arthritis Rheum 1996 39 9 1507 1518 10.1002/art.1780390910 8814062

[B8] Reed AM Pachman LM Hayford J Ober C Immunogenetic studies in families of children with juvenile dermatomyositis J Rheumatol 1998 25 5 1000 1002 9598907

[B9] Furuya T Hakoda M Higami K Ueda H Tsuchiya N Tokunaga K Kamatani N Kashiwazaki S Association of HLA class I and class II alleles with myositis in Japanese patients J Rheumatol 1998 25 6 1109 1114 9632072

[B10] Han X Zhai N Zhang Y LI J Liu J Song F Cehn H Association of HLA-DRB1 alleles and polymyositis/dermatomyositis in northen Chinese Chin J Microbiol Immunol 2003 23 3 225 227

[B11] Han X Zhai N Zhang Q Li J Liu J Du J Song F Association of HLA-DQB1 alleles and dermatomyositis/ polymyositis Chin J Med Genet 2002 19 4 322 323 12170471

[B12] Bohan A Peter JB Polymyositis and dermatomyositis (second of two parts) N Engl J Med 1975 292 8 403 407 10.1056/NEJM197502202920807 1089199

[B13] Bohan A Peter JB Polymyositis and dermatomyositis (first of two parts) N Engl J Med 1975 292 7 344 347 10.1056/NEJM197502132920706 1090839

[B14] Olerup O Aldener A Fogdell A HLA-DQB1 and -DQA1 typing by PCR amplification with sequence-specific primers (PCR-SSP) in 2 hours Tissue Antigens 1993 41 3 119 134 10.1111/j.1399-0039.1993.tb01991.x 8316943

[B15] Olerup O Zetterquist H HLA-DR typing by PCR amplification with sequence-specific primers (PCR-SSP) in 2 hours: an alternative to serological DR typing in clinical practice including donor-recipient matching in cadaveric transplantation Tissue Antigens 1992 39 5 225 235 10.1111/j.1399-0039.1992.tb01940.x 1357775

[B16] Stephens M Smith NJ Donnelly P A new statistical method for haplotype reconstruction from population data Am J Hum Genet 2001 68 4 978 989 10.1086/319501 11254454 PMC1275651

[B17] Engel AG Arahata K Emslie-Smith A Immune effector mechanisms in inflammatory myopathies Res Publ Assoc Res Nerv Ment Dis 1990 68 141 157 2158132

[B18] Robinson AB Reed AM Clinical features, pathogenesis and treatment of juvenile and adult dermatomyositis Nat Rev Rheumatol 2011 7 11 664 675 10.1038/nrrheum.2011.139 21947177

[B19] Shamim EA Rider LG Pandey JP O'Hanlon TP Jara LJ Samayoa EA Burgos-Vargas R Vazquez-Mellado J Alcocer-Varela J Salazar-Paramo M Kutzbach AG Malley JD Targoff IN Garcia-De la Torre I Miller FW Differences in idiopathic inflammatory myopathy phenotypes and genotypes between Mesoamerican Mestizos and North American Caucasians: ethnogeographic influences in the genetics and clinical expression of myositis Arthritis Rheum 2002 46 7 1885 1893 10.1002/art.10358 12124873

[B20] Chinoy H Salway F Fertig N Oddis CV Ollier WE Cooper RG Clinical, serological and HLA profiles in non-Caucasian UK idiopathic inflammatory myopathy Rheumatology (Oxford) 2009 48 5 591 592 19269956 10.1093/rheumatology/kep035

[B21] Marie I Hachulla E Levesque H Reumont G Ducrotte P Cailleux N Hatron PY Devulder B Courtois H Intravenous immunoglobulins as treatment of life threatening esophageal involvement in polymyositis and dermatomyositis J Rheumatol 1999 26 12 2706 2709 10606390

[B22] O'Hanlon TP Rider LG Mamyrova G Targoff IN Arnett FC Reveille JD Carrington M Gao X Oddis CV Morel PA Malley JD Malley K Shamim EA Chanock SJ Foster CB Bunch T Reed AM Love LA Miller FW HLA polymorphisms in African Americans with idiopathic inflammatory myopathy: allelic profiles distinguish patients with different clinical phenotypes and myositis autoantibodies Arthritis Rheum 2006 54 11 3670 3681 10.1002/art.22205 17075818

[B23] Yu KH Wu YJ Kuo CF See LC Shen YM Chang HC Luo SF Ho HH Chen IJ Survival analysis of patients with dermatomyositis and polymyositis: analysis of 192 Chinese cases Clin Rheumatol 2011 30 12 1595 1601 10.1007/s10067-011-1840-0 21915609

[B24] Kuo CF See LC Yu KH Chou IJ Chang HC Chiou MJ Luo SF Incidence, cancer risk and mortality of dermatomyositis and polymyositis in Taiwan: a nationwide population study Br J Dermatol 2011 165 6 1273 1279 10.1111/j.1365-2133.2011.10595.x 21895620

[B25] Chinoy H Lamb JA Ollier WE Cooper RG Recent advances in the immunogenetics of idiopathic inflammatory myopathy Arthritis Res Ther 2011 13 3 216 10.1186/ar3327 21658295 PMC3218878

[B26] Furuya T Hakoda M Tsuchiya N Kotake S Ichikawa N Nanke Y Nakajima A Takeuchi M Nishinarita M Kondo H Kawasaki A Kobayashi S Mimori T Tokunaga K Kamatani N Immunogenetic features in 120 Japanese patients with idiopathic inflammatory myopathy J Rheumatol 2004 31 9 1768 1774 15338498

[B27] Rider LG Shamim E Okada S Pandey JP Targoff IN O'Hanlon TP Kim HA Lim YS Han H Song YW Miller FW Genetic risk and protective factors for idiopathic inflammatory myopathy in Koreans and American whites: a tale of two loci Arthritis Rheum 1999 42 6 1285 1290 10.1002/1529-0131(199906)42:6<1285::AID-ANR28>3.0.CO;2-1 10366124

[B28] Chinoy H Salway F Fertig N Shephard N Tait BD Thomson W Isenberg DA Oddis CV Silman AJ Ollier WE Cooper RG In adult onset myositis, the presence of interstitial lung disease and myositis specific/associated antibodies are governed by HLA class II haplotype, rather than by myositis subtype Arthritis Res Ther 2006 8 1 R13 10.1186/ar1862 16507114 PMC1526560

[B29] Love LA Leff RL Fraser DD Targoff IN Dalakas M Plotz PH Miller FW A new approach to the classification of idiopathic inflammatory myopathy: myositis-specific autoantibodies define useful homogeneous patient groups Medicine (Baltimore) 1991 70 6 360 374 1659647 10.1097/00005792-199111000-00002

[B30] Hausmanowa-Petrusewicz I Kowalska-Oledzka E Miller FW Jarzabek-Chorzelska M Targoff IN Blaszczyk-Kostanecka M Jablonska S Clinical, serologic, and immunogenetic features in Polish patients with idiopathic inflammatory myopathies Arthritis Rheum 1997 40 7 1257 1266 10.1002/1529-0131(199707)40:7<1257::AID-ART10>3.0.CO;2-R 9214426

[B31] Feldman BM Rider LG Reed AM Pachman LM Juvenile dermatomyositis and other idiopathic inflammatory myopathies of childhood Lancet 2008 371 9631 2201 2212 10.1016/S0140-6736(08)60955-1 18586175

